# Essential qualities for dental practice: dental students' perspectives in Hong Kong

**DOI:** 10.3389/froh.2025.1603267

**Published:** 2025-06-17

**Authors:** Jasmine Cheuk Ying Ho, Hollis Haotian Chai, Ivy Guofang Sun, Michelle Zeping Huang, Edward Chin Man Lo, Chun Hung Chu

**Affiliations:** ^1^ Faculty of Dentistry, The University of Hong Kong, Hong Kong, Hong Kong SAR, China; ^2^ Department of English, The Hang Seng University of Hong Kong, Hong Kong, Hong Kong SAR, China

**Keywords:** students’ perspective, qualities of good dentists, qualities of successful dentist, key qualities for dental practice, dentist's attributes

## Abstract

**Objective:**

The objective of this quantitative study is to explore the perspectives of dental students in Hong Kong regarding the key qualities that define a good dentist and a successful dentist.

**Methods:**

A cross-sectional survey was conducted to Hong Kong dental students in 2024 using an anonymous questionnaire. The questionnaire basically consisted of four self-administered questions. The first two questions examined the qualities that dental students associate with “a successful dentist” and “a good dentist,” respectively. The third question focused on the qualities expected of their dentist, and the fourth question investigated the qualities that dental school training should emphasize. For each question, respondents were requested indicate three key or most essential qualities. They could write down or choose the qualities provided in the list with 24 qualities.

**Results:**

All 452 students in the dental school were invited and 399 students (88%) completed the survey. “Clinically competent” and “Good communication skills” and “Responsible/accountable” emerged as the top three qualities across the first three questions. They identified “Clinically competent”, “Good communication skills” and “knowledgeable” are three key qualities to be cultivated during training in dental school.

**Conclusions:**

Hong Kong dental students consider clinically competent, good communication skills, and being responsible/accountable are the key qualities for both good and successful dentists. These are also the key qualities they expected from their own dentist. Additionally, they emphasize the importance for dental schools to provide training that fosters clinical expertise, communication proficiency, and comprehensive knowledge to ensure graduates can deliver high-quality patient care.

**Significance:**

This survey offers important insights into the key qualities that dental students in Hong Kong believe are important for being a good and successful dentist. These findings can guide dental education to better prepare future professionals. Understanding these perspectives can help align educational goals with professional expectations, improving patient care and job satisfaction.

## 1 Introduction

Dental school curricula are often shaped by accreditation standards, which can differ widely between institutions. While many schools focus heavily on technical knowledge and clinical skills, this emphasis may sometimes leave less room for developing communication and interpersonal abilities, which are equally vital for comprehensive patient care (1). Although dental schools excel at producing technically skilled dentists, there is a growing recognition of the need to balance clinical expertise with other essential qualities that contribute to holistic and patient-centered practice.

Becoming a good and successful dentist is a common goal for most dental students. However, the specific qualities that define a “good” and “successful” dentist can vary considerably from one individual to another and among different stakeholders in the dental profession. Patients, for instance, tend to prioritize aspects such as personalized care, communication skills of a dentist, and overall satisfaction with their dental experience when evaluating their ideal dentist (2). A study exploring the factors influencing patients' selection of dentists revealed that competence, service quality (3, 4), effective communication, and interpersonal factors (5, 6) were significant attributes sought by patients. Similarly, another study on the attributes of a good dentist, as perceived by trainers at the Foundation Training in the United Kingdom (previously known as Vocational Training), yielded comparable outcomes, emphasizing the importance of competence, communication skills, and diagnostic acumen (7).

Understanding dental students' perspectives on the qualities of a good and successful dentist can shape dental education, helping schools tailor curricula and mentorship programs to better prepare future professionals. This insight identifies gaps in current practices, aligns training with real-world demands, and fosters a responsive educational environment. Engaging students ensures the development of well-rounded, competent dentists equipped to meet diverse patient needs while achieving personal and professional fulfillment.

While patients and trainers might focus on clinical and interpersonal skills, some dentists view success through a broader lens that extends beyond clinical competence to include financial and business acumen. Levin highlighted the significance of business knowledge and skills for establishing a successful dental practice (8). This disparity in perspectives raises intriguing questions about the alignment between being a good dentist and achieving success, posing a dilemma for aspiring dental students who must navigate these diverse expectations. It is essential to bridge the gap between educational outcomes and professional expectations, ensuring that dental graduates are well-prepared to meet the diverse needs of their patients while achieving success in their careers. The aim of this study is to delve into the perspectives of dental students regarding the attributes that define a good and successful dentist, thereby providing insights that can shape the future of dental education and practice.

## 2 Methods

A cross-sectional survey was conducted at the only dental school in Hong Kong in July 2024. The local Institutional Review Board approved this anonymous questionnaire survey. The dental school is highly subsidized by the government and offers a 6-year dental degree program with English as the medium of instruction. All 452 students studying in the school were invited to participate in the survey, and no specific exclusion criteria were set for this study.

Three research assistants conducted the survey immediately after the students attended their lectures in lecture halls or practical sessions in the simulation laboratory, with prior approval from the teachers in charge. The research assistants distributed the questionnaires to all students and asked them to complete the survey on the spot. They collected the completed questionnaires before the students left their classes.

In this questionnaire survey, demographic information including sex, age, and year of study was collected. The main content of the questionnaire consisted of four self-administered questions. The first two questions examined the qualities that dental students associate with “a successful dentist” and “a good dentist,” respectively. There was no description of what constitutes a “good dentist” or a “successful dentist” in the questionnaire. The third question focused on the qualities expected of their dentist, and the fourth question investigated the qualities that dental school training should emphasize.

For each question, respondents were requested to indicate the three key qualities. They could write down their answers or choose from a provided list of 24 qualities. These 23 qualities were derived from private dentists who were teaching part-time at the dental school. Two research assistants conducted small focus group discussions with four dentists in each group, using a non-probability institutional sampling method. There was no time limit for each discussion, but the researchers acted as facilitators to ensure that the discussions remained focused on the four questions. The researchers conducted five rounds of discussions until data saturation was reached.

**Figure 1:**
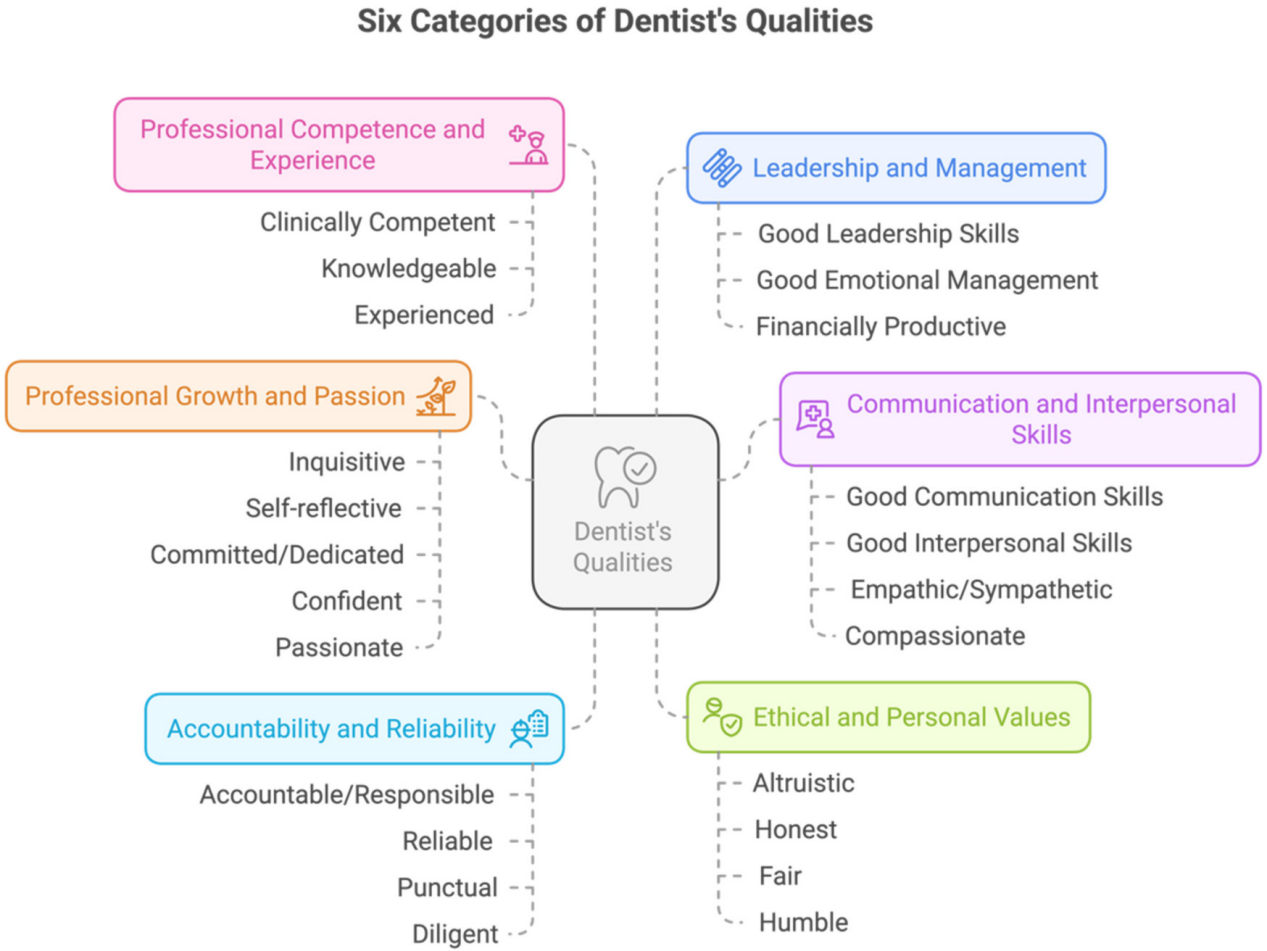
The six categories and qualities of dentists employed in the study.

Thematic analysis was performed to categorize the qualities (9). The researchers familiarized themselves with the qualities, generated initial codes, and searched and reviewed the data to define and name the identified characteristics into major groups. A total of six categories of dentist's qualities were developed (Figure 1).

Bivariate analysis was performed for key qualities chosen by at least 15% of the students based on their sex and year of study. Cochran's *Q* test was conducted to compare the selection of major dentist qualities among four questions.

## 3 Results

All 452 dental students (BDS 1–6) in Hong Kong were invited to participate, and 399 students completed the survey, resulting in a response rate of 88% (399/452). The main reason for non-response was absence from class (69%, 41/59). Out of the 390 participants who reported their sex, there were 242 (62%) female and 148 (38%) male. The mean age of the participants was 19.8 years (range 17–28). There were no invalid questionnaires collected. Nine students did not respond to the two demographic items on age and sex.

Table 1 highlights the five most important qualities students rated for a successful dentist, the three most important qualities for a good dentist, the three most important qualities students look for in a dentist when they are patients, and the qualities that students think dental schools should focus on when training dentists, respectively. Among the qualities, “clinical competence” was ranked as the most important quality across all four categories, with 277 (69%) responses for a successful dentist, 222 (56%) for a good dentist, 277 (69%) for expected qualities from their dentists, and 293 (73%) for qualities that dental training should focus on. Other highly endorsed qualities included “good communication skills,” “accountable/responsible,” “knowledgeable,” and “experienced.” The qualities that were least endorsed were “fair” and “altruism”.

**Table 1:** Essential qualities of dentists by student votes.

Key dentist qualities	For a successful dentist	For a good dentist	Of the student's own dentist	That dental training should focus
Clinically competent	**277**	**222**	**277**	**293**
Good communication skills	**180**	**171**	**130**	**174**
Accountable/responsible	**115**	**139**	**126**	**97**
Knowledgeable	**89**	**75**	97	**211**
Experienced	**70**	48	**127**	**67**
Financially productive	64	6	3	3
Reliable	58	61	**119**	27
Inquisitive	47	33	7	50
Good interpersonal skills	43	30	18	28
Sympathetic/empathetic	41	**90**	80	27
Honest	32	60	60	34
Confident	29	13	25	52
Self-reflective	25	25	8	32
Committed/dedicated	22	29	25	16
Good leadership skills	18	15	4	16
Compassionate	16	44	35	13
Diligent	16	11	4	14
Good emotional management	14	12	6	19
Passionate	14	17	11	16
Humble	9	10	6	8
Punctual	7	13	16	20
Altruism	5	1	0	1
Fair	1	2	3	0
Others (Please specify)	2 (Reputable)	1 (Hygienic)	1 (Discounted fee)	1 (Presentable)

Those bolded are the top five most highly rated qualities.

**Figure 2:**
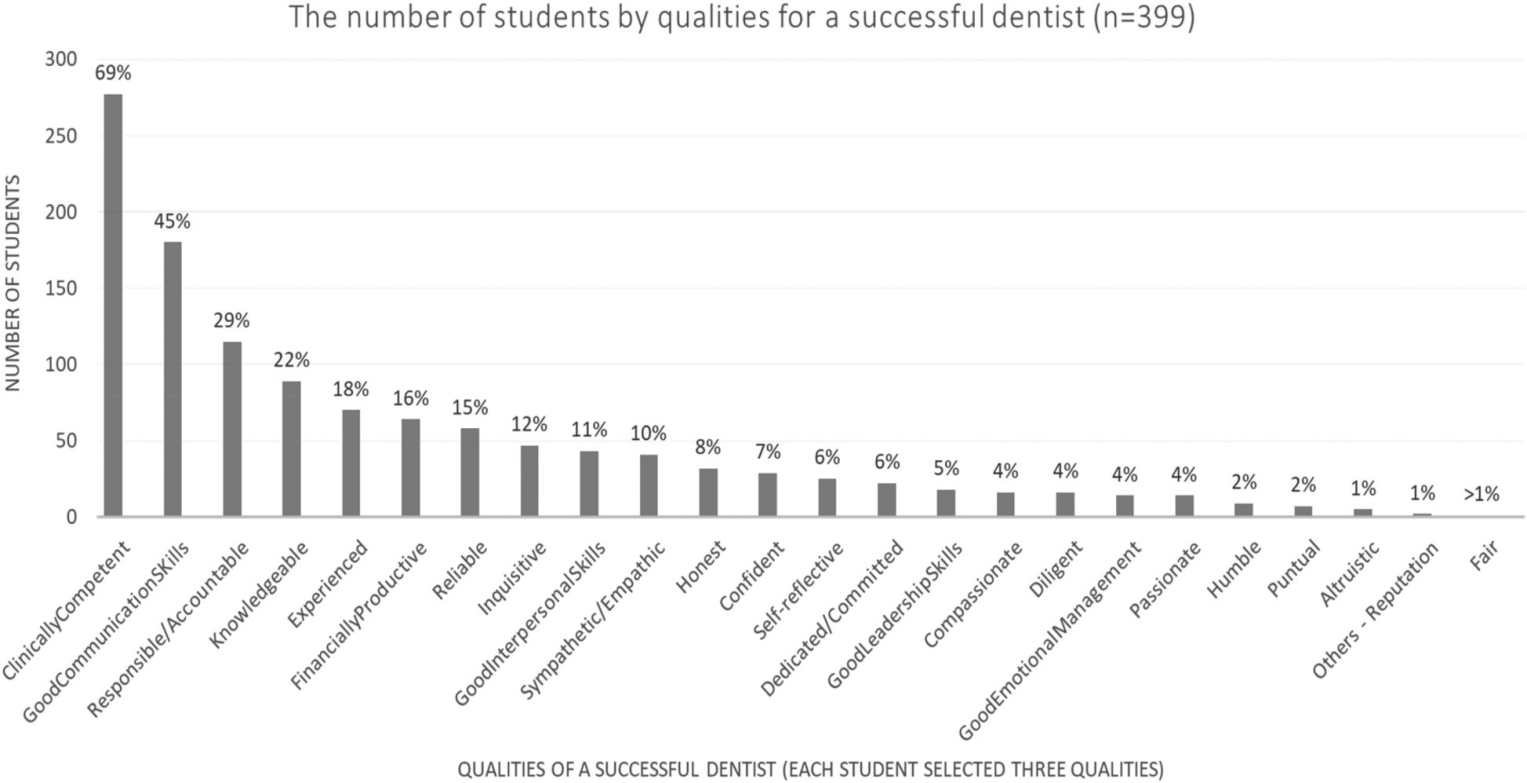
Distribution of essential qualities of a successful dentist as ranked by dental students.

**Figure 3:**
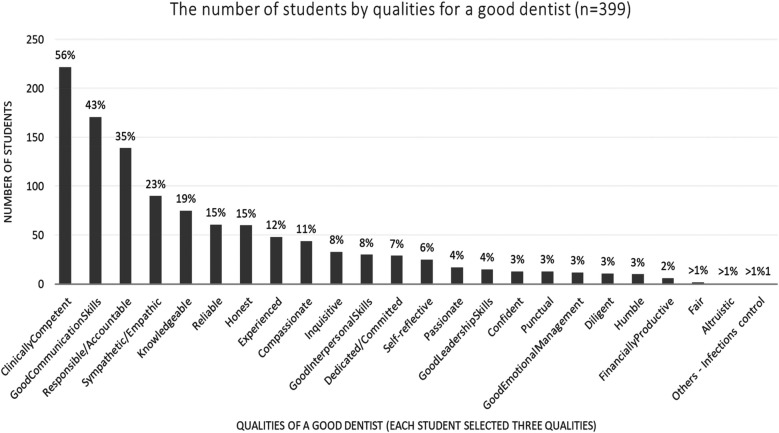
Distribution of essential qualities of a good dentist as ranked by dental students.

**Figure 4:**
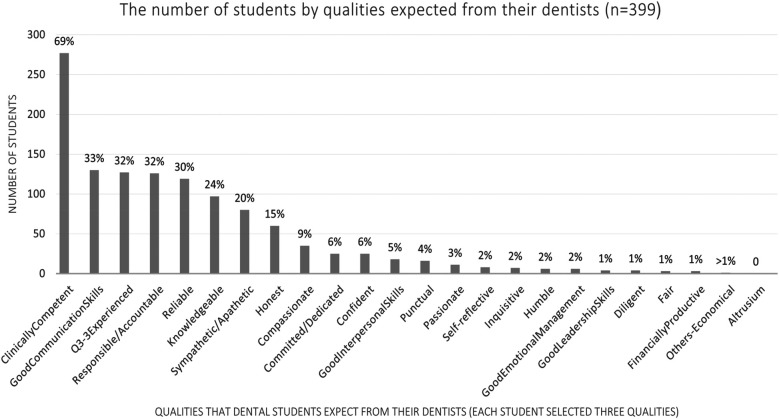
Student rankings of essential qualities expected from their dentists.

**Figure 5:**
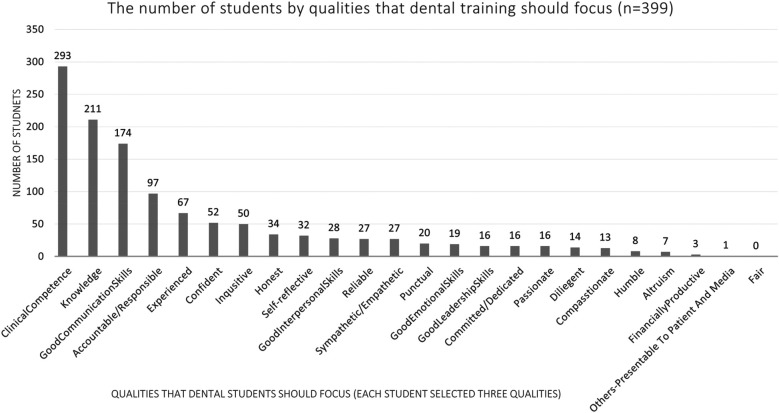
Student rankings of key qualities for university dental education focus.

The student rankings of the three most essential or key qualities of a good dentist and a successful dentist are shown in Figures 2, 3, respectively. Figure 4 illustrates the student rankings of qualities expected of a dentist when they are patients. Figure 5 displays the student rankings of the qualities that university education should focus on.

The essential qualities voted by students according to their sex and year of study for a successful dentist and a good dentist are shown in Tables 2, 3, respectively. Table 4 presents the student rankings of qualities expected of a dentist when they are patients. Table 5 shows the dentist qualities that university education should focus on, broken down by the sex and year of study of the students.

**Table 2:** Essential qualities of a successful dentist rated by students based on sex and year of study.

Category of dentist quality	Essential qualities for a successful dentist	Male (*n* = 148)	Female (*n* = 242)	Total (*n* = 390)	Year 1–2 (*n* = 146)	Year 3–6 (*n* = 253)	Total (*n* = 399)
Professional competence and experience	Clinical competence	95 (64%)	175 (72%)	270 (69%)	90 (62%)	187 (74%)	277 (70%)
	Knowledgeable	32 (22%)	56 (23%)	88 (23%)	31 (21%)	58 (23%)	89 (22%)
	Experienced	21 (14%)	47 (19%)	68 (17%)	25 (17%)	45 (18%)	70 (18%)
Communication and interpersonal skills	Good communication skills	68 (46%)	108 (45%)	176 (45%)	66 (45%)	114 (45%)	180 (45%)
	Good interpersonal skills	18 (12%)	25 (10%)	43 (11%)	12 (8%)	31 (12%)	43 (11%)
	Empathic/sympathetic	18 (12%)	23 (10%)	41 (11%)	21 (14%)	20 (8%)	41 (10%)
	Compassionate	5 (3%)	11 (5%)	16 (4%)	5 (3%)	11 (4%)	16 (4%)
Accountability and reliability	Accountable/responsible	42 (28%)	67 (28%)	109 (28%)	42 (29%)	73 (29%)	115 (29%)
	Reliable	17 (11%)	38 (16%)	55 (14%)	26 (18%)	32 (13%)	58 (15%)
	Diligent	10 (7%)	6 (2%)	16 (4%)	8 (5%)	8 (3%)	16 (4%)
	Punctual	0	7 (3%)	7 (2%)	3 (2%)	4 (2%)	7 (2%)
Leadership and management	Financially productive	30 (20%)	32 (13%)	62 (16%)	33 (23%)	31 (12%)	64 (16%)
	Good leadership skills	7 (5%)	11 (5%)	18 (5%)	5 (3%)	13 (5%)	18 (5%)
	Good emotional management	5 (3%)	9 (4%)	14 (4%)	4 (3%)	10 (4%)	14 (4%)
Professional growth and passion	Inquisitive	13 (9%)	34 (14%)	47 (12%)	15 (10%)	32 (13%)	47 (12%)
	Confident	17 (11%)	12 (5%)	29 (7%)	13 (9%)	16 (6%)	29 (7%)
	Self-reflective	11 (7%)	13 (5%)	24 (6%)	7 (5%)	18 (7%)	25 (6%)
	Committed/dedicated	8 (5%)	14 (6%)	22 (6%)	10 (7%)	12 (5%)	22 (6%)
	Passionate	6 (4%)	8 (3%)	14 (4%)	4 (3%)	10 (4%)	14 (4%)
Ethical values and personal values	Honest	11 (7%)	21 (9%)	32 (8%)	11 (8%)	21 (8%)	32 (8%)
	Humble	5 (3%)	4 (2%)	9 (2%)	3 (2%)	6 (2%)	9 (2%)
	Altruistic	2 (1%)	3 (1%)	5 (1%)	2 (1%)	3 (1%)	5 (1%)
	Fair	1 (1%)	0	1 (1%)	0	1 (1%)	1 (1%)

**Table 3:** Essential qualities of a good dentist rated by students based on sex and year of study.

Category of dentist quality	Essential qualities for a successful dentist	Male (*n* = 148)	Female (*n* = 242)	Total (*n* = 390)	Year 1–2 (*n* = 146)	Year 3–6 (*n* = 253)	Total (*n* = 399)
Professional competence and experience	Clinical Competence	88 (59%)	144 (60%)	232 (59%)	89 (61%)	147 (58%)	236 (59%)
	Knowledgeable	34 (23%)	47 (19%)	81 (21%)	36 (25%)	46 (18%)	82 (21%)
	Experienced	18 (12%)	33 (14%)	51 (12%)	23 (16%)	28 (11%)	51 (13%)
Communication and interpersonal skills	Good communication skills	55 (37%)	120 (50%)	175 (45%)	72 (49%)	106 (42%)	178 (45%)
	Good interpersonal skills	34 (23%)	57 (24%)	91 (23%)	34 (23%)	59 (23%)	93 (23%)
	Empathic/sympathetic	18 (12%)	27 (11%)	45 (12%)	10 (7%)	35 (14%)	45 (11%)
	Compassionate	15 (10%)	16 (7%)	31 (8%)	10 (7%)	21 (8%)	31 (8%)
Accountability and reliability	Accountable/responsible	42 (28%)	98 (40%)	140 (26%)	53 (36%)	91 (36%)	144 (26%)
	Reliable	19 (13%)	41 (17%)	60 (15%)	21 (14%)	40 (16%)	61 (15%)
	Diligent	7 (5%)	7 (3%)	14 (4%)	5 (3%)	9 (4%)	14 (4%)
	Punctual	7 (5%)	6 (2%)	13 (3%)	9 (6%)	6 (2%)	15 (4%)
Leadership and management	Financially productive	8 (5%)	9 (4%)	17 (4%)	8 (5%)	9 (4%)	17 (4%)
	Good leadership skills	7 (5%)	5 (2%)	12 (3%)	2 (1%)	10 (4%)	12 (3%)
	Good emotional management	1 (1%)	5 (2%)	6 (2%)	3 (2%)	3 (1%)	6 (2%)
Professional growth and passion	Inquisitive	17 (11%)	19 (8%)	36 (9%)	12 (8%)	25 (10%)	37 (9%)
	Confident	11 (7%)	21 (9%)	32 (8%)	7 (5%)	25 (10%)	32 (8%)
	Self-reflective	12 (8%)	12 (5%)	24 (6%)	9 (6%)	17 (7%)	26 (7%)
	Committed/dedicated	7 (5%)	10 (4%)	17 (4%)	3 (2%)	15 (6%)	18 (5%)
	Passionate	8 (5%)	7 (3%)	15 (4%)	6 (4%)	10 (4%)	16 (4%)
Ethical values and personal values	Honest	26 (18%)	31 (13%)	57 (15%)	17 (12%)	44 (17%)	61 (15%)
	Humble	5 (3%)	6 (2%)	11 (3%)	3 (2%)	8 (3%)	11 (3%)
	Altruistic	0	1 (1%)	1 (1%)	1 (1%)	0	1 (1%)
	Fair	1 (1%)	0	1 (1%)	0	2 (1%)	2 (1%)

**Table 4:** Essential qualities students look for in their own dentists by sex and year of study.

Category of dentist quality	Essential qualities for a successful dentist	Male (*n* = 148)	Female (*n* = 242)	Total (*n* = 390)	Year 1–2 (*n* = 146)	Year 3–6 (*n* = 253)	Total (*n* = 399)
Professional competence and experience	Clinical Competence	105 (71%)	164 (68%)	269 (69%)	98 (67%)	179 (71%)	277 (70%)
	Knowledgeable	49 (33%)	76 (31%)	125 (32%)	40 (27%)	87 (34%)	127 (32%)
	Experienced	37 (25%)	59 (24%)	96 (25%)	41 (28%)	56 (22%)	97 (24%)
Communication and interpersonal skills	Good communication skills	39 (26%)	88 (36%)	127 (33%)	50 (34%)	80 (32%)	130 (33%)
	Good interpersonal skills	35 (24%)	43 (18%)	78 (20%)	26 (18%)	54 (21%)	80 (20%)
	Empathic/sympathetic	4 (3%)	31 (13%)	35 (9%)	10 (7%)	25 (10%)	35 (9%)
	Compassionate	5 (3%)	13 (5%)	18 (5%)	7 (5%)	11 (4%)	18 (5%)
Accountability and reliability	Accountable/responsible	41 (28%)	82 (34%)	123 (32%)	48 (33%)	78 (31%)	126 (32%)
	Reliable	42 (28%)	72 (30%)	114 (29%)	38 (26%)	81 (32%)	119 (30%)
	Diligent	8 (5%)	7 (3%)	15 (4%)	6 (4%)	10 (4%)	16 (4%)
	Punctual	3 (2%)	1 (1%)	4 (1%)	2 (1%)	2 (1%)	4 (1%)
Leadership and management	Financially productive	4 (3%)	2 (1%)	6 (2%)	2 (1%)	4 (2%)	6 (2%)
	Good leadership skills	2 (1%)	2 (1%)	4 (1%)	3 (2%)	1 (1%)	4 (1%)
	Good emotional management	0 (0)	3 (1%)	3 (1%)	1 (1%)	2 (1%)	3 (1%)
Professional growth and passion	Inquisitive	11 (7%)	14 (6%)	25 (6%)	8 (5%)	17 (7%)	25 (6%)
	Confident	15 (10%)	10 (4%)	25 (6%)	17 (12%)	8 (3%)	25 (6%)
	Self-reflective	2 (1%)	9 (4%)	11 (3%)	5 (3%)	6 (2%)	11 (3%)
	Committed/dedicated	4 (3%)	4 (2%)	8 (2%)	2 (1%)	6 (2%)	8 (2%)
	Passionate	3 (2%)	4 (2%)	7 (2%)	5 (3%)	2 (1%)	7 (2%)
Ethical values and personal values	Honest	25 (17%)	33 (14%)	58 (15%)	22 (15%)	38 (15%)	60 (15%)
	Humble	3 (2%)	3 (1%)	6 (2%)	2 (1%)	4 (2%)	6 (2%)
	Altruistic	3 (2%)	0	3 (1%)	2 (1%)	1 (1%)	3 (1%)
	Fair	0	0	0	0	0	0

**Table 5:** Essential qualities for dental education emphasis as rated by students by sex and year of study.

Category of dentist quality	Essential qualities for a successful dentist	Male (*n* = 148)	Female (*n* = 242)	Total (*n* = 390)	Year 1–2 (*n* = 146)	Year 3–6 (*n* = 253)	Total (*n* = 399)
Professional competence and experience	Clinical competence	107 (72%)	178 (74%)	285 (73%)	103 (71%)	190 (75%)	293 (74)
	Knowledgeable	75 (51%)	130 (54%)	205 (53%)	76 (52%)	135 (53%)	211 (53%)
	Experienced	24 (16%)	42 (17%)	66 (17%)	20 (14%)	47 (19%)	67 (17%)
Communication and interpersonal skills	Good communication skills	61 (41%)	109 (45%)	170 (44%)	63 (43%)	111 (44%)	174 (44%)
	Good interpersonal skills	11 (7%)	17 (7%)	28 (7%)	8 (6%)	20 (8%)	28 (7%)
	Empathic/sympathetic	11 (7%)	16 (7%)	27 (7%)	9 (6%)	18 (7%)	27 (7%)
	Compassionate	4 (3%)	9 (4%)	13 (3%)	3 (2%)	10 (47%)	13 (3%)
Accountability and reliability	Accountable/responsible	35 (24%)	60 (25%)	95 (24%)	43 (30%)	54 (21%)	97 (24%)
	Reliable	12 (8%)	14 (6%)	26 (7%)	15 (10%)	12 (5%)	27 (7%)
	Diligent	9 (6%)	10 (4%)	19 (5%)	10 (7%)	9 (4%)	19 (5%)
	Punctual	4 (3%)	10 (4%)	14 (4%)	7 (5%)	7 (3%)	14 (4%)
Leadership and management	Financially productive	10 (7%)	9 (4%)	19 (5%)	9 (6%)	10 (4%)	19 (5%)
	Good leadership skills	5 (3%)	10 (4%)	15 (4%)	6 (4%)	10 (4%)	16 (4%)
	Good emotional management	3 (2%)	0 (0)	3 (>1%)	0 (0)	3 (1%)	3 (>1%)
Professional growth and passion	Inquisitive	15 (10%)	34 (14%)	49 (13%)	15 (10%)	35 (14%)	50 (13%)
	Confident	9 (6%)	22 (9%)	31 (8%)	8 (6%)	24 (9%)	32 (8%)
	Self-reflective	11 (7%)	9 (4%)	20 (5%)	8 (6%)	12 (5%)	20 (5%)
	Committed/dedicated	4 (3%)	11 (5%)	15 (4%)	9 (6%)	7 (3%)	16 (4%)
	Passionate	8 (5%)	8 (3%)	16 (4%)	9 (6%)	7 (3%)	16 (4%)
Ethical values and personal values	Honest	16 (11%)	17 (7%)	33 (8%)	7 (5%)	27 (11%)	34 (9%)
	Humble	5 (3%)	3 (1%)	8 (2%)	5 (3%)	3 (1%)	8 (2%)
	Altruistic	3 (2%)	4 (2%)	7 (2%)	2 (1%)	5 (2%)	7 (2%)
	Fair	0 (0)	0 (0)	0 (0)	0 (0)	0 (0)	0 (0)

Bivariate analysis was performed for key qualities chosen by at least 15% of the students based on their sex and year of study (Table 6). There was no significant difference between sexes in rating key dentist qualities in terms of a successful dentist and qualities that dental education should focus on. However, there were significant differences in the qualities “good communication skills” and “accountable/responsible” ranked by female and male students in terms of a good dentist. There were more female students consider good communication skills and accountability/responsibility to be the important qualities of a good dentist than male students do, both with the *P*-value of 0.02. Moreover, more female students considered good communication skills to be the important quality of their dentists when they were patients with a *P*-value of 0.04. On the other hand, when we see the difference between the senior and junior students regarding the important qualities for dental care, there were more senior students (years 3–6) than junior students (years 1–2) considered clinical competence to be the important quality for a successful dentist with a *P*-value of 0.01. Conversely, more junior students rated financial productivity as the important quality for a successful dentist with *P*-value (0.01).

**Table 6:** Bivariate analysis of major dentist qualities rated by at least 15% of students, by sex and year of study.

Dentist qualities	Sex (*n* = 390)			Year of study (*n* = 399)			
	Male (*n* = 148)	Female (*n* = 242)	*p* value	Year 1–2 (*n* = 146)	Year 3–6 (*n* = 253)		*p* value
For a successful dentist							
Clinical Competence	95 (64%)	175 (72%)	0.09	90 (62%)		187 (74%)	**0**.01*
Knowledgeable	32 (22%)	56 (23%)	0.73	31 (21%)		58 (23)	0.70
Experienced	21 (14%)	47 (19%)	0.19	25 (17%)		45 (18%)	0.87
Good communication skills	68 (46%)	108 (45%)	0.80	66 (45%)		114 (45%)	0.98
Accountable/responsible	42 (28%)	67 (28%)	0.88	42 (29%)		73 (29%)	0.99
Financially productive	30 (20%)	32 (13%)	0.07	33 (23%)		31 (12%)	**0**.01*
For a good dentist							
Clinical competence	88 (59%)	144 (60%)	0.99	89 (61%)		147 (58%)	0.58
Good communication skills	55 (37%)	120 (50%)	**0**.02*	72 (49%)		106 (42%)	0.15
Accountable/responsible	42 (28%)	98 (40%)	**0**.02*	53 (36%)		91 (36%)	0.95
Sympathetic/empathetic	34 (23%)	57 (24%)	0.90	34 (23%)		59 (23%)	0.99
Knowledgeable	34 (23%)	47 (19%)	0.40	36 (25%)		46 (18%)	0.12
Of the students own dentists							
Clinical competence	105 (71%)	164 (68%)	0.51	98 (67%)		179 (70%)	0.45
Good communication skills	39 (26%)	88 (36%)	**0**.04*	50 (34%)		80 (32%)	0.59
Experienced	49 (33%)	76 (31%)	0.73	40 (27%)		87 (34%)	0.15
Accountable/responsible	41 (28%)	82 (34%)	0.20	48 (33%)		78 (31%)	0.67
Reliable	42 (28%)	72 (30%)	0.77	38 (26%)		81 (32%)	0.21
Knowledgeable	37 (25%)	59 (24%)	0.89	41 (28%)		56 (22%)	0.18
Sympathetic/empathetic	35 (24%)	43 (18%)	0.16	26 (18%)		54 (21%)	0.40
That dental training should focus							
Clinical competence	107 (72%)	178 (74%)	0.74	103 (71%)		190 (75%)	0.38
Knowledgeable	75 (51%)	130 (54%)	0.53	76 (52%)		135 (53%)	0.86
Good communication skills	61 (41%)	109 (45%)	0.44	63 (43%)		111 (44%)	0.93
Accountable/responsible	35 (23%)	60 (25%)	0.78	43 (29$)		54 (21%)	0.06
Experienced	24 (16%)	42 (17%)	0.76	20 (14%)		47 (19%)	0.22

* Significant difference.

Cochran's *Q* test was conducted to compare the selection of major dentist qualities among four questions (Table 7). The results revealed significant differences (*p* < 0.001) in how dental students prioritized qualities across the four questions. “Clinical competence” was most frequently selected for a successful dentist (Q1, 69%), their own dentist (Q3, 69%), and dental training focus (Q4, 70%), but less so for a good dentist (Q2, 56%). “Knowledgeable” was prioritized for training focus (Q4, 53%) over other questions (19%–24%). “Experienced” was emphasized for their own dentists (Q3, 32%) but less for other contexts. “Good communication skills” were valued similarly in Q1 (45%), Q2 (43%), and Q4 (44%) but less for their own dentists (Q3, 33%). “Financial productivity” (Q1, 16%) and “reliability” (Q3, 30%) showed context-dependent prioritization, while “sympathetic” and “empathetic” were more linked to good dentists (Q2, 23%) and expectations from their dentists (Q3, 20%) than training (Q4: 7%).

**Table 7:** Comparison of major dentist qualities among four questions.

Dentists’ qualities	Yes (%)	*p* value*	Pairwise comparisons
Clinical competence (*n* = 398)		<0.001	
Q1- for a successful dentist	277 (69%)		Q1 = Q3 = Q4
Q2- for a good dentist	222 (56%)		
Q3- of the students own dentists	277 (69%)		
Q4- that dental training should focus	293 (70%)		
Knowledgeable (*n* = 398)		<0.001	
Q1- for a successful dentist	89 (22%)		Q1 = Q2 = Q3
Q2- for a good dentist	75 (19%)		
Q3- of the students own dentists	97 (24%)		
Q4- that dental training should focus	211 (53%)		
Experienced (*n* = 398)		<0.001	
Q1- for a successful dentist	70 (18%)		Q1 = Q2 = Q4
Q2- for a good dentist	48 (12%)		
Q3- of the students own dentists	127 (32%)		
Q4- that dental training should focus	67 (17%)		
Good communication skills (*n* = 398)		<0.001	
Q1- for a successful dentist	180 (45%)		Q1 = Q2 = Q4
Q2- for a good dentist	171 (43%)		
Q3- of the students own dentists	130 (33%)		
Q4- that dental training should focus	174 (44%)		
Accountable/responsible (*n* = 398)		<0.001	
Q1- for a successful dentist	115 (29%)		Q2 = Q3 Q1 = Q3 Q1 = Q4
Q2- for a good dentist	139 (35%)		
Q3- of the students own dentists	126 (32%)		
Q4- that dental training should focus	97 (24%)		
Financially productive (*n* = 398)		<0.001	
Q1- for a successful dentist	64 (16%)		Q2 = Q3 = Q4
Q2- for a good dentist	6 (2%)		
Q3- of the students own dentists	3 (1%)		
Q4- that dental training should focus	3 (1%)		
Sympathetic/Empathetic (*n* = 398)		<0.001	
Q1- for a successful dentist	41 (10%)		Q1 = Q4 Q2 = Q3
Q2- for a good dentist	90 (23%)		
Q3- of the students own dentists	80 (20%)		
Q4- that dental training should focus	27 (7%)		
Reliable (*n* = 398)		<0.001	
Q1- for a successful dentist	58 (15%)		Q1 = Q2 Q1 = Q4
Q2- for a good dentist	61 (15%)		
Q3- of the students own dentists	119 (30%)		
Q4- that dental training should focus	27 (7%)		

* Cochran's *Q* test (with significant difference, *p* > 0.05).

## 4 Discussion

The field of dentistry demands a unique blend of skills and attributes to ensure high-quality patient care and professional success. This study explored the perspectives of dental students in Hong Kong regarding the essential qualities of a good and successful dentist, the qualities they expect in their own dentists, and the attributes they believe should be emphasized during dental training. By analyzing the findings of a survey conducted among dental students, we can gain valuable insights into how future dental professionals perceive their roles and responsibilities. To ensure unbiased responses, the questionnaire deliberately avoided defining terms such as “good dentist” or “successful dentist”, and students were not briefed on these concepts beforehand. This approach was implemented to preserve data integrity and capture authentic perceptions. Participants were also given the flexibility to choose from predefined options or add their own free-text responses, striking a balance between structured data collection and open-ended feedback. While this format emphasized quantitative analysis, it may have limited the expression of spontaneous perspectives—a limitation that could be addressed in future qualitative studies aimed at exploring students' unfiltered experiences in greater depth.

This study achieved a very high response rate from the students, likely due to the strong support and promotion from their teachers during lectures and simulation courses. Teachers encouraged student participation and facilitated the immediate distribution, completion, and collection of the questionnaires. The investigator's direct invitation to students also helped increase their willingness to respond. The cross-sectional survey was designed to be anonymous to encourage honest and uninfluenced responses (10).

This study used a simple questionnaire with only four questions, offering several advantages. Firstly, it tends to achieve a higher response rate because it is less time-consuming, making respondents more likely to complete it (11). It also helps to keep the survey focused on the most important questions, ensuring that the primary objectives of the study are addressed (12). Additionally, a shorter questionnaire reduces respondent fatigue, leading to more accurate answers (13). This approach is also cost-effective as it is usually less expensive to administer and analyze (14). In addition, the use of standardized questions offered several advantages. Data analysis, especially statistical analysis, is straightforward because the results are consistent and measurable (14). This consistency reduces variability in the responses, making it easier to compare data across different respondents.

The survey consisted of four self-administered questions focusing on the qualities associated with a successful dentist, a good dentist, the qualities expected of their dentist, and the qualities that dental school training should emphasize. Respondents were asked to indicate the three most essential qualities for each question by either selecting from a provided list of 23 qualities or providing their answers under the “others” option. The order of choices in the questionnaire was structured based on the categorization of qualities, ensuring no inherent relationship between the ranking of choices presented and the order reflected in the respondents' selections. Analysis of the data indicates that the respondents' decisions were not influenced by the sequence in which the choices were presented, confirming that the order of choices had no measurable impact on their decision-making process.

However, there are also limitations to consider. With fewer questions, the depth of information collected is limited, potentially missing out on valuable insights. The lack of context provided by a short questionnaire may not fully capture the respondents' answers, leading to a less comprehensive understanding of their perspectives. Furthermore, key aspects or variables might be omitted due to the brevity of the questionnaire, which could affect the overall quality and completeness of the data collected. Closed-ended questions do not allow respondents to elaborate on their answers, which can limit the depth and richness of the data collected.

Our findings clearly highlight the qualities dental students in Hong Kong deem crucial for success in dentistry, with clinical competence consistently emerging as the most essential quality across all four questions in the survey. Clinical competence is widely recognized as a fundamental requirement for all dental practitioners. This aligns with the Code of Professional Discipline for the Guidance of Dental Practitioners in Hong Kong (15), which mandates that all practicing dentists must possess essential and specialized skills to deliver professional oral care to the community. The students' emphasis on clinical competence reflects their understanding of its critical role in ensuring effective patient care and professional success, reinforcing the standards set by regulatory bodies in the field of dentistry. Clinical competence encompasses the multifaceted ability to enhance cognitive and psychomotor skills, as well as to cultivate evolving behaviors and attitudes that develop in tandem with increasing technical proficiency through meaningful patient interactions (16). This competence is essential for ensuring the effective and safe performance of dental procedures, involving a combination of technical skills, diagnostic acumen, and the practical application of theoretical knowledge in real-world settings.

Our findings also showed that students identified good communication skills as one of the top qualities for a successful dentist and a good dentist. This finding aligns with a similar survey conducted in the United Kingdom, which highlighted the importance of communication skills in great dentists among young dentists (17). The two studies shared similar results as the instincts of dental students and early-career dentists were well-founded. While technical knowledge and clinical competence are undeniably important, good communication emerges as the predominant factor in effective dental practice. Students also indicated good communication skills as an important quality expected from their dentists. Good communication between dentists and patients involves a collaborative exchange of ideas regarding clinical care goals and professional recommendations, aimed at identifying the optimal treatment plan for the patient's oral health (18). Numerous studies exploring patient expectations of dentists, which highlight the importance of both technical expertise and interpersonal communication skills in dental practice (19–21). While clinical competence ensures the delivery of accurate diagnoses and treatments, interpersonal communication skills are equally vital in fostering trust, enhancing patient compliance, and improving overall treatment outcomes (22). Students anticipate that their dentists will not only possess the necessary technical skills, but also communicate effectively, providing clear explanations of procedures and addressing concerns with empathy. This dual emphasis on clinical competence and communication reflects a holistic understanding of what constitutes effective and patient-centered dental care, underscoring the need for dental education programs to integrate both technical and interpersonal skill development to meet the expectations of future patients.

Responsibility/Accountability was rated as one of the top three qualities of a good dentist and a successful dentist. In medical and general healthcare, responsibility entails legal accountability and ethical or moral obligations to uphold and promote the patient's well-being (23). This includes adhering to established standards of care, maintaining patient confidentiality, and being accountable for one's actions. According to the model of professionalism in dentistry, responsibility/accountability along with vocation, and altruism are the clearly observable characteristics, behaviors, or qualities that define professionalism in a person's actions or interactions (24). The findings of this study showed that students highly valued responsibility and accountability as the crucial attribute of a successful and a good dentist, demonstrating their understanding of their core principles of professionalism in dentistry and the significance they place on these qualities within the profession. Students expect dentists to exhibit integrity, prioritize patient well-being, and take responsibility for their actions.

The study showed that male and female students rated important dentist qualities similarly. However, more female than male students prioritized “good communication skills” and “accountability/responsibility” as important qualities of a good dentist. Additionally, female students placed greater emphasis on good communication skills than male students when considering the qualities they value in their own dentists as patients. These findings were in line with existing literature which highlights the tendency of female healthcare professionals (25–27) and female patients (28, 29) to prioritize empathetic communication skills in clinical practice, demonstrating clear gender differences in communication styles. Female patients, for instance, tend to ask more questions, seek detailed information, want to receive more counselling and preventive services, and engage in more participatory visits compared to male patients (25, 28, 29). Similarly, female physicians are more likely to engage in information sharing, discuss psychosocial topics, build partnerships, and encourage patient participation than their male counterparts (25–27). These findings underscore the importance of recognizing gender differences in communication styles within oral healthcare. In some countries, female doctors might be perceived as more comfortable asking deeper or more personal questions due to social and cultural norms, communication styles, and patient comfort levels. These factors can lead to greater trust and rapport, making it easier for female doctors to discuss sensitive issues with their patients. On the other hand, senior students valued “clinical competence” as a key quality for a successful dentist more than junior students did. As students advance in their education and gain practical experience, they develop a greater appreciation for the technical and clinical skills required in dentistry.

The findings of this study shed light on the perspectives of dental students regarding the attributes they value in their dentists. Notably, the results reveal that most dental students prioritize clinical competence, followed by communication skills. This aligns partially with existing literature on patients' perspectives, which emphasize communication as the top priority, followed by clinical competence (3). The slight divergence in the order of priorities between dental students and patients offers an interesting point of reflection on how professional training and patient expectations intersect. The prioritization of clinical competence by dental students is consistent with their role as emerging professionals who are deeply immersed in the technical and practical aspects of dentistry. For dental students, the ability of their dentists to demonstrate expertise, precision, and proficiency in clinical procedures is likely viewed as foundational to effective patient care. However, while patients tend to prioritize communication skills above all else, dental students place it as their second priority. This difference may stem from the unique context of dental education. As students, their primary focus is on acquiring and mastering clinical skills, which may lead them to place greater emphasis on competence. Communication, while still highly valued, may be perceived as a complementary skill rather than the foremost priority during their training.

Regarding the results of how dental students prioritized qualities across the four questions. The findings highlight contextual nuances in students' perceptions. Clinical competence and knowledge were prioritized for successful dentists and professional training, aligning with technical proficiency as a career cornerstone. However, qualities such as empathy, reliability, and accountability were more strongly associated with “good dentists” and personal expectations, suggesting students distinguish between technical success and interpersonal virtue. The lower emphasis on communication and empathy in training (Q4) vs. personal expectations (Q3) implies a potential gap in curricular focus. Financial productivity, though modestly linked to “success,” was deemphasized in training and ethical contexts, reflecting tensions between professional success and traditional care values. These findings underscore the need for dental education to balance technical and interpersonal competencies to align with students' holistic ideals of professionalism.

## 5 Implications for dental education

The findings of this survey can guide dental educators in developing curricula and training programs that address the needs and expectations of future dental professionals, ultimately improving patient care and job satisfaction in the field of dentistry.

First, the emphasis on clinical competence highlights the importance of rigorous practical training in dental schools. Students must be provided with ample opportunities to practice and hone their skills in a supervised environment. Simulation labs, hands-on workshops, and clinical rotations are integral components of dental education, as evidenced by previous studies (30–32). These experiential learning methods have been positively perceived by students and have been shown to enhance their knowledge, technical skills, and confidence in clinical practice (30–32). Moreover, implementing a continuous assessment approach involves triangulating data from multiple sources of feedback, using various methods and assessments over time to offer a comprehensive and precise representation of student competence (33). Continual assessment and feedback from experienced faculty can assist students in honing their techniques and meeting the profession's high standards.

Second, it is recommended that dental curricula integrate training in communication skills, encompassing patient interviews, counseling, and conflict resolution. Effective development of communication skills goes beyond theoretical coursework; it necessitates practical application and introspection. Lectures, interactive workshops, and role-playing scenarios have been identified as successful methods for enhancing students' communication abilities (34–36). Furthermore, self-assessment and feedback from peers and instructors offer valuable perspectives on areas that require enhancement (37). By emphasizing communication skills in dental education, institutions can graduate professionals who excel not only in technical proficiency but also in delivering compassionate and patient-centered care.

Third, the emphasis on responsibility and accountability highlights the need for robust training on professionalism within the dental education. The dental literature has described professionalism as an important component of competence (38). While definitions commonly encompass ethics, jurisprudence, and appropriate behaviour toward patients and dental team members, the emphasis on other aspects varies. The UK General Dental Council (GDC) mandates the assessment of professionalism throughout undergraduate dental programs and continues to incorporate it into learning outcomes (39). Methods for evaluating knowledge and skills have been well-documented (40, 41), and there is a substantial body of literature on assessing professionalism within medical education (42–44). Additionally, educators are tasked with establishing clear expectations to guide student development (45). Therefore, it is essential for dental educators to have a precise understanding of what “professionalism” entails in the context of dentistry to effectively teach and assess it. Instilling a sense of responsibility and accountability in dental students is essential for fostering a culture of integrity and excellence. Ethical training should be a fundamental part of the dental curriculum, with courses on professional ethics, legal responsibilities, and patient rights. Case studies and ethical dilemmas can be used to stimulate critical thinking and discussion, helping students understand the complexities of real-world practice.

## 6 Conclusions

Hong Kong dental students consider clinically competent, good communication skills, and being responsible/accountable as the key qualities for both good and successful dentists. These are also the key qualities they expected from their own dentist. They also expected a dental school to offering training so that the students would become clinically competent, having good communication and knowledgeable to serve their patients. This survey offers important insights into the key qualities that dental students in Hong Kong believe are important for being a good and successful dentist. These findings can guide dental education to better prepare future professionals. Understanding these perspectives can help align educational goals with professional expectations, improving patient care and job satisfaction.

## Data Availability

The original contributions presented in the study are included in the article/Supplementary Material, further inquiries can be directed to the corresponding author.

## References

[1] Timofe MP Albu S. Quality management in dental care: patients’ perspectives on communication. a qualitative study. Clujul Med. (2016) 89(2):287. 10.15386/cjmed-532 27152082 PMC4849389

[2] Sbaraini A Carter SM Evans RW Blinkhorn A. Experiences of dental care: what do patients value? BMC Health Serv Res. (2012) 12:177. 10.1186/1472-6963-12-177 22726888 PMC3407476

[3] Ungureanu MI Mocean F. What do patients take into account when they choose their dentist? Implications for quality improvement. Patient Prefer Adherence. (2015) 9:1715–20. 10.2147/PPA.S94310 26664071 PMC4669916

[4] Kim MJ Damiano PC Hand J Denehy GE Cobb DS Qian F. Consumers’ choice of dentists: how and why people choose dental school faculty members as their oral health care providers. J Dent Educ. (2012) 76(6):695–704. 10.1002/j.0022-0337.2012.76.6.tb05303.x 22659697

[5] St Louis BL Firestone AR Johnston W Shanker S Vig KW. Prospective patients rate practice factors: development of a questionnaire. Am J Orthod Dentofacial Orthop. (2011) 139(2):235–41. 10.1016/j.ajodo.2009.06.028 21300253

[6] Bedair TM Thompson S Gupta C Beck FM Firestone AR. Orthodontists’ opinions of factors affecting patients’ choice of orthodontic practices. Am J Orthod Dentofacial Orthop. (2010) 138(1):6.e1–7. discussion 6–7. 10.1016/j.ajodo.2010.02.020 20620820

[7] Buck D Malik S Murphy N Patel V Singh S Syed B What makes a good dentist and do recent trainees make the grade? The views of vocational trainers. Br Dent J. (2000) 189(10):563–6. 10.1038/sj.bdj.4800829a 11128260

[8] Levin RP. Succeeding as a new dentist. J Am Dent Assoc. (2014) 145(3):290–1. 10.14219/jada.2014.6 24583897

[9] Chai HH Gao SS Chen KJ Duangthip D Lo ECM Chu CH. A concise review on qualitative research in dentistry. Int J Environ Res Public Health. (2021) 18(3). 10.3390/ijerph18030942 PMC7908600 33499023

[10] Chu CH Lo EC. Patients’ satisfaction with dental services provided by a university in Hong Kong. Int Dent J. (1999) 49(1):53–9. 10.1111/j.1875-595X.1999.tb00508.x 10887474

[11] Goodfellow LT. An overview of survey research. Respir Care. (2023) 68(9):1309–13. 10.4187/respcare.11041 37072162 PMC10468179

[12] Jain S Dubey S Jain S. Designing and validation of questionnaire. Int Dent Med J Adv Res. (2016) 2(1):1–3. 10.15713/ins.idmjar.39

[13] Lund E Gram IT. Response rate according to title and length of questionnaire. Scand J Soc Med. (1998) 26(2):154–60. 10.1177/14034948980260020401 9658516

[14] Ballinger C Davey C. Designing a questionnaire: an overview. Br J Occup Ther. (1998) 61(12):547–50. 10.1177/030802269806101204

[15] Hong Kong TDCoH. Code of professional discipline for the guidance of dental practitioners in Hong Kong. (2019). Available at: https://www.dchk.org.hk/pdf/code.pdf (Accessed May 05, 2025).

[16] Marchan SM Coldero LG Smith WAJ. An evaluation of the relationship between clinical requirements and tests of competence in a competency-based curriculum in dentistry. BMC Med Educ. (2023) 23(1):585. 10.1186/s12909-023-04438-3 37596584 PMC10439671

[17] London General Dental Council. Preparing for practice: dental team learning outcomes for registration 2015. (2015). Available at: https://www.gdc-uk.org/docs/default-source/education-and-cpd/preparing-for-practice-(revised-2015).pdf?sfvrsn=81d58c49_2 (Accessed May 05, 2025).

[18] Ho JCY Chai HH Lo ECM Huang MZ Chu CH. Strategies for effective dentist-patient communication: a literature review. Patient Prefer Adherence. (2024) 18:1385–94. 10.2147/PPA.S465221 38974679 PMC11225999

[19] Khan N. A survey of patients opinion for business and professional factors affecting private dental practices in Riyadh, Saudi Arabia. J Dow Univ Health Sci (JDUHS). (2007) 1(1):3–9. Available at: https://jduhs.com/index.php/jduhs/article/view/1246

[20] Edwards DT Shroff B Lindauer SJ Fowler CE Tufekci E. Media advertising effects on consumer perception of orthodontic treatment quality. Angle Orthod. (2008) 78(5):771–7. 10.2319/083106-357.1 18298216

[21] Lamprecht R Struppek J Heydecke G Reissmann DR. Patients’ criteria for choosing a dentist: comparison between a university-based setting and private dental practices. J Oral Rehabil. (2020) 47(8):1023–30. 10.1111/joor.12995 32428967

[22] Ho JCY Chai HH Luo BW Lo ECM Huang MZ Chu CH. An overview of dentist-patient communication in quality dental care. Dent J (Basel). (2025) 13(1):31. 10.3390/dj13010031 39851608 PMC11763373

[23] Freukel DA Lurie Y. Responsibility and liability in health care: some differences between dentistry and medicine. Med Law. (2002) 21(3):605–15. Available at: https://www.researchgate.net/publication/11031113 12437206

[24] Zijlstra-Shaw S Roberts TE Robinson PG. Perceptions of professionalism in dentistry—a qualitative study. Br Dent J. (2013) 215(9):E18. 10.1038/sj.bdj.2013.1048 24201649

[25] Bertakis KD Franks P Epstein RM. Patient-centered communication in primary care: physician and patient gender and gender concordance. J Womens Health (Larchmt). (2009) 18(4):539–45. 10.1089/jwh.2008.0969 19361322

[26] Roter DL Hall JA. Physician gender and patient-centered communication: a critical review of empirical research. Annu Rev Public Health. (2004) 25:497–519. 10.1146/annurev.publhealth.25.101802.123134 15015932

[27] Roter DL Hall JA Aoki Y. Physician gender effects in medical communication: a meta-analytic review. JAMA. (2002) 288(6):756–64. 10.1001/jama.288.6.756 12169083

[28] Hall JA Roter DL. Patient gender and communication with physicians: results of a community-based study. Womens Health. (1995) 1(1):77–95. 9373374

[29] Elliott MN Lehrman WG Beckett MK Goldstein E Hambarsoomian K Giordano LA. Gender differences in patients’ perceptions of inpatient care. Health Serv Res. (2012) 47(4):1482–501. 10.1111/j.1475-6773.2012.01389.x 22375827 PMC3401395

[30] Inkabi SE Bernstein J. Enhancing knowledge, skills, and confidence of oral health professionals through head simulator training: a perceived benefit. J Educ Learn. (2024) 13(3):17–27. 10.5539/jel.v13n3p17

[31] Deeb GR Johnson A Bondarew M Carrico C Laskin D Deeb JG. How effective is a dental workshop at improving the knowledge and confidence of medical students in the management of dental emergencies? J Med Educ Curric Dev. (2016) 3:91–6. 10.4137/JMECD.S39992 PMC5736274 29349314

[32] Harrington C Robinson F Mallery SR. Clinical teaching in dentistry: evaluating a clinical oral pathology rotation while looking to the future of dental education. J Dent Educ. (2023) 87(7):1016–21. 10.1002/jdd.13206 36999553

[33] van der Vleuten C Sluijsmans D Joosten-ten Brinke D. Competence Assessment as Learner Support in Education. New Jersey: Springer (2017).

[34] El Tantawi MM Abdelaziz H AbdelRaheem AS Mahrous AA. Using peer-assisted learning and role-playing to teach generic skills to dental students: the health care simulation model. J Dent Educ. (2014) 78(1):85–97. 10.1002/j.0022-0337.2014.78.1.tb05660.x 24385528

[35] Alvarez S Schultz JH. A communication-focused curriculum for dental students—an experiential training approach. BMC Med Educ. (2018) 18(1):55. 10.1186/s12909-018-1174-6 29587740 PMC5872386

[36] Hannah A Millichamp CJ Ayers KM. A communication skills course for undergraduate dental students. J Dent Educ. (2004) 68(9):970–7. 10.1002/j.0022-0337.2004.68.9.tb03846.x 15342658

[37] Quick KK. The role of self- and peer assessment in dental students’ reflective practice using standardized patient encounters. J Dent Educ. (2016) 80(8):924–9. 10.1002/j.0022-0337.2016.80.8.tb06172.x 27480703

[38] Cowpe J Plasschaert A Harzer W Vinkka-Puhakka H Walmsley AD. Profile and competences for the graduating European dentist—update 2009. Eur J Dent Educ. (2010) 14(4):193–202. 10.1111/j.1600-0579.2009.00609.x 20946246

[39] Hanks S Ranauta A Johnson I Bateman H Nasseripour M Neville P. Professionalism and dental education: in search of a shared understanding. Br Dent J. (2022) 232(7):470–4. 10.1038/s41415-022-4094-0 35396431

[40] Brown GA Bull J Pendlebury M. Assessing Student Learning in Higher Education. London: Routledge (2013).

[41] Norman GR van der Vleuten CP Newble DI. International Handbook of Research in Medical Education. New York: Springer Science & Business Media (2012).

[42] Lynch DC Surdyk PM Eiser AR. Assessing professionalism: a review of the literature. Med Teach. (2004) 26(4):366–73. 10.1080/01421590410001696434 15203852

[43] Stern DT. Measuring Medical Professionalism. Oxford: Oxford University Press (2006). Available at: https://www.researchgate.net/profile/Mohammadreza-Hojat/publication/291294283_Measuring_specific_elements_of_Professionalism_Empathy_Teamwork_and_Lifelong_learning/links/578cf83508ae254b1de869fe/Measuring-specificelements-of-Professionalism-Empathy-Teamwork-and-Lifelong-learning.pdf (Accessed May 10, 2025).

[44] Veloski JJ Fields SK Boex JR Blank LL. Measuring professionalism: a review of studies with instruments reported in the literature between 1982 and 2002. Acad Med. (2005) 80(4):366–70. 10.1097/00001888-200504000-00014 15793022

[45] Stern DT Papadakis M. The developing physician—becoming a professional. N Engl J Med. (2006) 355(17):1794–9. 10.1056/NEJMra054783 17065641

